# The impact of ground losses on estimations of lightning-induced voltage with dispersive soil parameters

**DOI:** 10.1038/s41598-025-08379-3

**Published:** 2025-07-06

**Authors:** Toqa Faisal, Sahar. S. Kaddah, Taghreed Said, Mohammad E. M. Rizk

**Affiliations:** 1 Electrical Power and Machines Department, Faculty of Engineering, Mansoura University, Mansoura, Egypt; 2 Electrical Engineering Department, Higher Technological Institute, 10th of Ramada City, Egypt

**Keywords:** Electromagnetic field, Lightning induced voltage, Frequency-dependent soil parameters, Ground losses, Engineering, Electrical and electronic engineering

## Abstract

In areas with frequent lightning activity, studying lightning induced voltage on overhead distribution lines is crucial to improve the line performance under nearby lightning strikes. This article studies the impact of ground losses in the transmission line model on lighting-induced voltage using an analytical approach. The soil characteristics was represented using fixed and frequency dependent soil models. Firstly, the lightning electromagnetic fields have been computed considering the influence of finite ground conductivity using Cooray-Rubinstein model. Afterwards, the lightning-induced voltage has been computed using Agrawal coupling model in frequency domain. The results show that incorporating ground losses in the transmission line model has a considerable effect on lightning-induced voltages at different velocities. The influence of velocity was evaluated at: 40 m/μs, 120 m/μs, and 200 m/μs respectively. It was found that the influence of ground losses on the peak value of LIVs is more significant at 40 m/μs. Since the median value of lightning velocities is 120 m/μs, it is deduced that the influence of ground losses is more pronounced at lower velocities as it causes an increase in the magnitude of lightning-induced voltage. The effect of incorporating ground losses in the transmission line model on lightning-induced voltage is also evaluated at various values of transmission line height. The effect of transmission line height was examined at 6 m and 10 m respectively. It was found that the effect of ground losses is greater at the height of 10 m at the midpoint of the transmission line. Consequently, as the height of the transmission line decreases, the influence of ground losses also decreases. However, the opposite occurs at positions far from the midpoint, where the influence of ground losses increases with lower transmission line heights. The impact of incorporating ground losses in the transmission line model on lightning-induced voltage is examined at different distances between the lightning channel and the transmission line. The effect of these distances was examined at values of 50 m and 100 m respectively. It was found that the influence of ground losses diminishes at distances of 100 m and above. Furthermore, lightning induced voltage magnitude with frequency dependent soil model is lower than with fixed soil model.

## Introduction

The investigation of lightning-induced voltage (LIV) on overhead lines is substantial to avoid outage due to the resultant flashovers on the insulators^1^. Due to the rising reliability requirements for power delivery, the issue of LIV on overhead lines and underground power cables has been reexamined in recent years^2^.

The evaluation of LIVs requires the electromagnetic field distribution along the line. The authors evaluated the electromagnetic field emitted by dipole^3^. The authors created method for calculating the electromagnetic radiation of a dipole when it is placed over a half-space that conducts electricity to a limited degree. They achieved this by solving Maxwell’s equations for both the dipole and the conducing half-space, while considering the boundary conditions at the interface between the air and the ground^4^. The resulting equations used in this study are expressed in the frequency domain as slowly converging integrals known as Sommerfeld integrals. In^5,6^, numerical methods are presented for the finite-difference time-domain method (FDTD) and the finite-element method (FEM). Lightning electromagnetic field (LEMF) should be computed along the line, as these electromagnetic fields serve as the excitation terms in field-to-line coupling models. Therefore, computations require extensive CPU memory and running time. The authors also described various advanced approximations to this formulation namely the Norton and the Bannister methods^7^. The Norton and Bannister approaches have their validity limits defined based on the distance between the lightning channel and a specific point, as well as the frequency involved.

The computed electromagnetic field consists of horizontal electric, vertical electric, and azimuthal magnetic components. The authors have shown that the intensity of the vertical electric field (VEF) component and the azimuthal magnetic field (AMF) can be computed with rational approximation assuming that the ground is perfectly conducting at distances from the lightning channel not exceeding a few kilometers using dipole method^8^. The ground’s limited conductivity has a greater impact on the lightning’s horizontal electric field (HEF) component. The HEF plays a significant role in the computations of LIVs^8,9^. The author suggested computing the HEF at the surface of a finitely conducting ground using formula of surface impedance of ground^9^. The AMF is used to calculate the HEF at ground level using the surface impedance formula. Another approach is outlined which the HEF is divided into two components the HEF above perfectly conducting ground and a correction factor for AMF which represents the effect of the finite ground^10^. The Cooray-Rubinstein formula (CR) is used in this work to calculate the HEF at ground level^11,12^.

The HEF component and LIV calculation are influenced by soil parameters. The soil parameters such as conductivity *σ*_*g*_ and permittivity ${\varepsilon }_{rg}$ are both affected by frequency. Due to the skin effect, the phenomenon leads to the concentration of alternating current near the surface of conductors and soil particles, resulting in higher current density at the surface and an overall increase in soil conductivity^13^. Furthermore, at higher frequencies, ion mobility within the soil is enhanced, leading to an additional increase in conductivity. The higher frequency also results in a decrease in soil permittivity, as the rapid changes in the electric field prevent proper alignment of soil molecules. Conversely, at lower frequencies, permittivity is higher due to molecular alignment. A realistic model of soil parameters improves the evaluation accuracy of the HEF and LIV on overhead lines^13^. Many articles developed different models for frequency-dependent soil parameters as Scott Model, Smith-Longmire Model, Messier Model, Visacro-Portela Model, Visacro-Alipio Model, and Alipio-Visacro^6,14–17^. Alipio-Visacro Model and Smith-Longmire Model are adopted for the investigations in this research^6,15^.

The general theory of coupling, with particular importance to LIV calculations, describes LEMF interact with transmission line (TL)^18–20^. Initially, we present field-to-TL coupling equations of Agrawal obtained using TL approximation^12,21^ and discuss the fundamental assumptions of the TL theory^21^. Next, the field-to-TL coupling equations is derived for the scenario where a single-wire line is positioned above a conducting ground that represents both perfect conductivity and loss^7,20,21^.

Considering ground losses impact in TL model on LIV needs further investigation, particularly with dispersive soil in which soil is represented using frequency dependent parameters. Effect of ground losses in field to TL coupling model is considered in case of fixed and dispersive soil parameters. This study was conducted for different cases of the main factors influencing LIV, which are lightning velocity *v*, TL tower height *h* and distance between lightning strike and TL tower *d.* This study has been implemented in the frequency domain.

## Mathematical models

### Computation of lightning electromagnetic fields

**Fig. 1:**
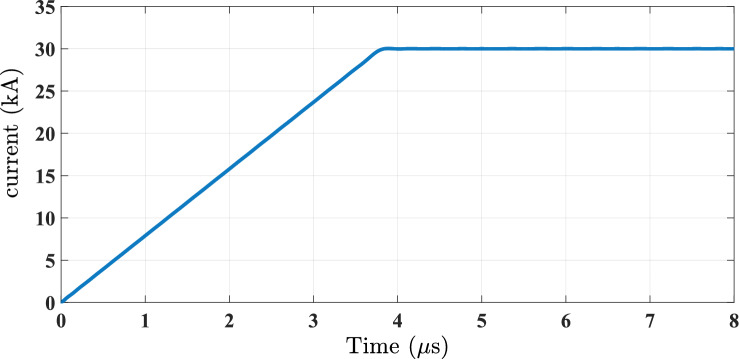
Trapezoidal current waveform for the typical first stroke; *T* = 3.8 µs and *I*_*o*_ = 30 kA.

Various models are widely utilized to describe lightning return-stroke current waveforms, including the Heidler function, the double exponential function, and the trapezoidal waveform function. Lightning return-stroke currents are frequently represented using the Heidler function. The Heidler function provides a smooth and continuous waveform without discontinuities^22,23^. The double exponential function has been considered for simulating lightning return-stroke currents. However, it can exhibit problems related to the discontinuities of its derivatives at onset time^23^. The trapezoidal shape is another simple approach to representing return-stroke currents ^24^. It accurately approximates the rising portions and peak value of the lightning current waveform, providing a straightforward representation of lightning characteristic^23,24^. The trapezoidal waveform is easy to find its Laplace transform, but it is difficult to represent the Heidler function within the frequency domain. Figure 1 represents trapezoidal current waveform and shows that the peak value of 30 kA and the rise time of 3.8 *µ*s are considered for the typical first negative return-stroke^24^.

Trapezoidal waveform in frequency domain is represented by Eq. (1):

1 $$I(j\omega )=\frac{{I}_{o}}{T{(j\omega )}^{2}}-\frac{{I}_{o}}{T{(j\omega )}^{2}}{e}^{-j\omega T}$$

where *I*_*o*_ is the peak value of lightning stroke current, and *T* is the rise time of current waveform.

Calculating the electromagnetic field in a specific scenario can be quite extensive and requires knowledge of the underlying physics and mathematical techniques. Maxwell’s equations, a collection of partial differential equations, are commonly used to describe the electromagnetic field and establish a relationship between the electric and magnetic fields.

**Fig. 2:**
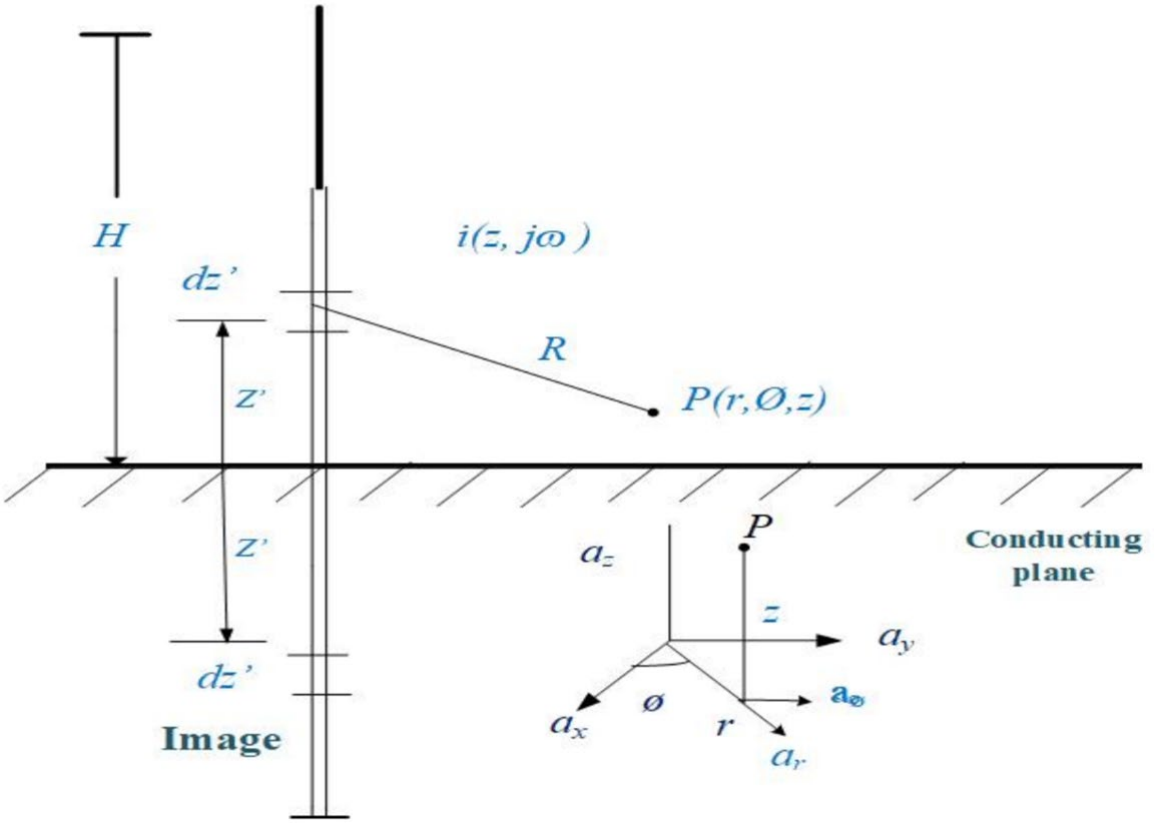
Geometry for electric dipole geometry for calculation of LEMFs distribution.

Figure 2 illustrates the geometry in cylindrical coordinates used to formulate HEF, VEF, and AMF. The ground is assumed to be a perfectly conducting surface for Eqs. (2) and (3) in the frequency domain, which represent the VEF intensity *E*_*z*_ and the AMF *H*_*Ø*_*,* respectively.

2 $$\begin{aligned} E_{z} (r,z,j\omega ) & = \frac{1}{{4\uppi \varepsilon _{o} }}\left( {\int\limits_{{ - H}}^{H} {\frac{{2\left( {z - z^{\prime} } \right)^{2} - r^{2} }}{{R^{5} }}\frac{1}{{j\omega }}I\left( {z^{\prime} ,j\omega } \right)e^{{ - \frac{{j\omega R}}{c}}} dz^{\prime} } } \right. + \int\limits_{{ - H}}^{H} {\frac{{2\left( {z - z^{\prime} } \right)^{2} - r^{2} }}{{cR^{4} }}I(z^{\prime} ,j\omega )e^{{ - \frac{{j\omega R}}{c}}} dz^{\prime}} \\ & \quad - \int\limits_{{ - H}}^{H} {\frac{{r^{2} }}{{c^{2} R^{3} }}j\omega I\left( {z^{\prime},j\omega } \right)e^{{ - \frac{{j\omega R}}{c}}} dz^{\prime}} \\ \end{aligned}$$

3 $$H_{\emptyset } (r,z,j\omega ) = \frac{1}{{4\uppi }}\left( {\int\limits_{{ - H}}^{H} {\frac{r}{{R^{3} }}I\left( {z^{\prime},j\omega } \right)e^{{ - \frac{{j\omega R}}{c}}} dz^{\prime}} + \int\limits_{{ - H}}^{H} {\frac{r}{{cR^{2} }}j\omega I\left( {z^{\prime},j\omega } \right)e^{{ - \frac{{j\omega R}}{c}}} dz^{\prime}} } \right)$$

where *R* = $\sqrt{{r}^{2}-{\left(z-{z}{\prime}\right)}^{2}}$ represents the distance between the lightning channel and the location where the field is being calculated. *I* (${z}{\prime}$, *jω*) represents the current distribution along the lightning channel in the frequency domain. The height of the lightning channel is represented by *H*, which is approximately 2500 m above the ground surface in this investigation, as referenced in^25^, the speed of light is represented as *c*. The horizontal distance between the lightning channel and the location where the electromagnetic field is calculated is represented as *r*. Additionally, the height of the point where the field is being calculated above the ground is denoted as *z*.

*E*_*rp*_ (*r, z, jω*) represents the HEF induced by the lightning channel at height* z* and distance *r*, assuming the ground to be a perfect conductor^26^. *E*_*rp*_ is represented by Eq. (4) in the frequency domain.

4 $$\begin{aligned} E_{{rp}} (r,z,j\omega ) & = \frac{1}{{4\uppi \varepsilon _{o} }}\left( {\int\limits_{{ - H}}^{H} {\frac{{3r\left( {z - z^{\prime}} \right)}}{{R^{5} }}\frac{1}{{J\omega }}I\left( {z^{\prime},j\omega } \right)e^{{ - \frac{{j\omega R}}{c}}} dz^{\prime}} + \int\limits_{{ - H}}^{H} {\frac{{3r\left( {z - z^{\prime}} \right)}}{{cR^{4} }}I\left( {z^{\prime},j\omega } \right)e^{{\frac{{j\omega R}}{c}}} dz^{\prime}} } \right. \\ & \quad + \int\limits_{{ - H}}^{H} {\frac{{r\left( {z - z^{\prime}} \right)}}{{c^{2} R^{3} }}j\omega e^{{ - \frac{{j\omega R}}{c}}} dz^{\prime}} \\ \end{aligned}$$

Cooray has demonstrated the HEF at lossy ground surface that can be calculate using Eq. (5):

5 $$E_{{rg}} (r,0,j\omega ) = H_{\emptyset } (r,0,j\omega )\frac{{c\mu _{o} }}{{\sqrt {\varepsilon _{{rg}} + \frac{{\sigma _{g} }}{{j\omega \varepsilon _{o} }}} }}$$

where ${\sigma }_{g}$ is soil conductivity, ${\varepsilon }_{rg}$ is soil permittivity and ${\varepsilon }_{o}$ is free space permittivity.

The main assumptions of the Rubinstein approximation are *σ*_*g*_ $\gg$ *ω* ${\varepsilon }_{o}{\varepsilon }_{rg}^{2}$ and the HEF at ground surface is not impacted by the finite ground conductivity. The Rubinstein approximation can be reformulated more generally to generate Eq. (6), which is known as the CR formula when *σ*_*g*_ $\gg$ *ω* ${\varepsilon }_{o}{\varepsilon }_{rg}$ low frequency condition is not fulfilled. The CR formula provides a term that represents the surface impedance of the ground* Z*_*CR*_ beneath the TL where the LIV is calculated. This term, expressed as $\left(c{\mu }_{o}/\sqrt{{\varepsilon }_{rg}+ \frac{{\sigma }_{g}}{j\omega {\varepsilon }_{o}}}\right)$, depends on the electrical properties of the soil, including ${\varepsilon }_{rg}$ and ${\sigma }_{g}$. The CR formula can be expressed as follows:

6 $$E_{r} (r,z,j\omega ) = E_{{rp}} (r,z,j\omega ) - E_{{rg}} (r,0,j\omega )$$

The CR formula provides accurate results when the line is not too close to the lightning stroke and the ground resistivity is not excessively high. For instance, it works correctly for ground resistivities of 100, 1000, and 10,000 Ω m at observation distances greater than 50, 200, and 500 m, respectively^27^. However, these limitations apply only to the HEF at ground level. For HEF above ground, the electrostatic field’s contribution must be considered. Since the electrostatic field is largely unaffected by ground resistivity, the HEF above ground is less influenced by ground currents compared to the HEF at ground level^27^.

### Field to line coupling equation

The Agrawal model utilizes TL theory to compute the LIV on TL, which can be analyzed in the frequency domain or time domain. The model assumes that the transmission line is a single line above the ground (perfectly conducting surface) on a specific height *h* and that the lightning channel is a vertical dipole antenna. The single-wire line is represented by inductance *L*ʹ per unit length, capacitance *C*ʹ per-unit-length and the exciting electric field from the lightning channel is implemented by distributed voltage sources positioned along the line in the lossless equivalent circuit of a single-wire overhead line as defined by Agrawal model^28^. The model can be extended to deal with wire and ground losses. Ground losses impact both the propagation of surges along the line and the HEF component. Since these ground losses depend on soil parameters, it is thoughtful to consider not only the fixed parameters of soil but also the frequency-dependent parameters for dispersive soil. The wire losses are frequency-dependent due to skin effects. The wire and the ground losses were ignored in the TL model for LIV calculation by the previous research^6,24,28^.

### Theory of transmission line

Power networks typically span several kilometers, which is significantly greater than the minimum wavelengths associated with lightning electromagnetic pulse (LEMP). In fact, large parts of the LEMP frequency spectrum reach up to several megahertz, corresponding to minimum wavelengths of approximately 100 m or less^28,29^. The typical TL transverse dimensions, on the other hand, are significantly smaller than the minimum wavelength of LEMP.

The following points are the basic presumptions of the TL theory^28,29^:

1. LEMP propagates along the axis of the line only. This occurs when the cross section of the line is small electrically.
2. The ground provides the return path for currents in overhead conductors, ensuring that the net current at any cross-section is zero. This assumption prioritizes “TL mode” currents and neglects “antenna-mode” currents, which are insignificant near the line’s ends. Even though antenna-mode currents are present along the line, the near-symmetry of the ground plane ensures that TL mode currents are the primary contributors
3. The LEMP produced by lightning-induced currents along the line is confined to the transverse plane and is oriented perpendicular to the line’s axis.

### The field-To-TL coupling equations

Agrawal’s Field-to-TL equations are shown below assuming the TL approximation applied.

7 $$\frac{{dV_{s} \left( x \right)}}{{dx}} + j\omega L^{\prime}I(x) = E_{x}^{e} \left( {x,0,h} \right)$$

8 $$\frac{{dI\left( x \right)}}{{dx}} + j\omega C^{\prime}V_{s} (x) = 0$$

where $L^{\prime}$ is known as the longitudinal inductance per-unit-length, $C^{\prime}$ is known as transverse capacitance per unit length, *I*(x) is symbol of induced current through the wire, and $V_{s}$ is scattered voltage.

**Fig. 3:**
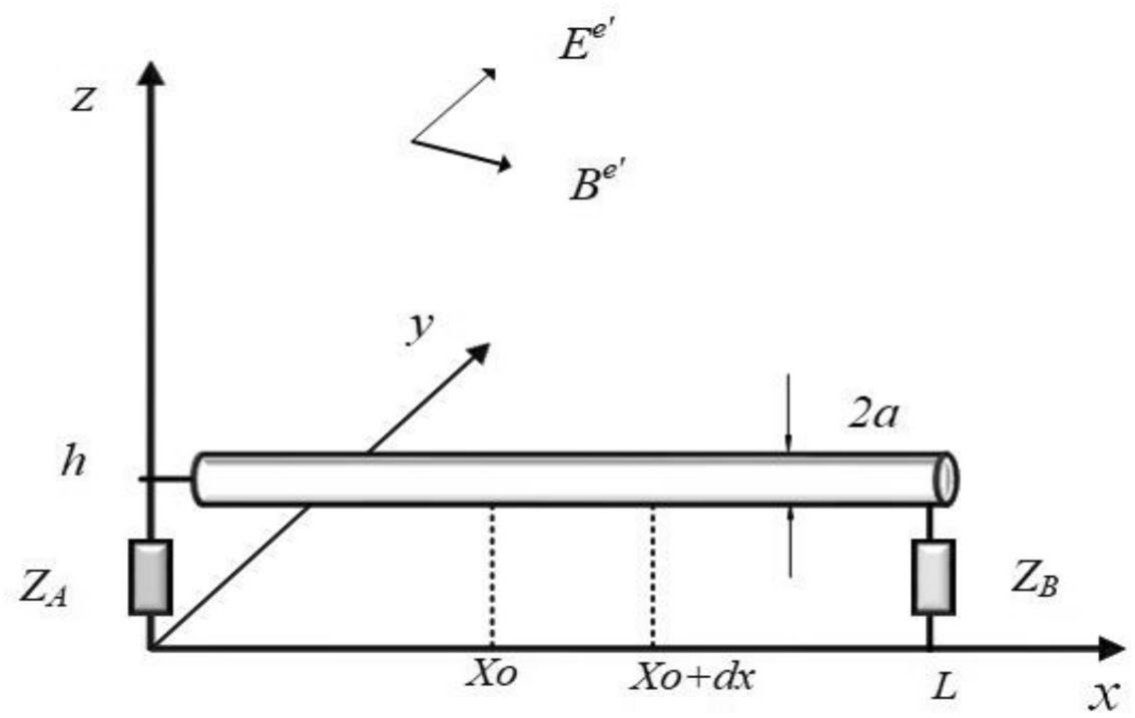
Terminating the single-wire line above ground with impedances *Z*_*A*_ and *Z*_*B*_.

For the termination impedances *Z*_*A*_ and *Z*_*B*_ shown in Fig. 3, the boundary condition is expressed in terms of the scattered voltage and total current.

9 $${V}_{s}(0) = -Z_{A} I (0) +{\int }_{0}^{h}{E}_{z}^{e}(0,0, z) dz$$

10 $${V}_{s}(L) = Z_{B} I (L) +{\int }_{0}^{h}{E}_{z}^{e}(L,0, z) dz$$

**Fig. 4:**
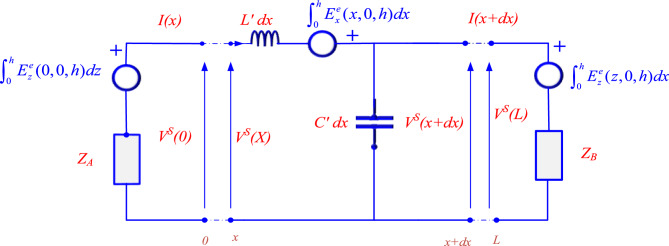
The circuit model proposed by Agrawal et al. for a lossless single-wire overhead line energized by an electromagnetic field.

Figure 4 shows the equivalent circuit of the model described by Eqs. (7–10). To satisfy boundary conditions in Eqs. (9) and (10), two lumped voltage sources are incorporated at the terminations of the line in this model. Furthermore, distributed voltage sources are used along the line to represent the exciting electric field that acts as the forcing function and is tangential to the line conductor. This model solely focuses on the electric field component of the exciting electromagnetic field. The coupling equations do not include the exciting magnetic field as a distinct source term.

When calculating LIVs, losses in the wire and ground can be considered in theory. The electromagnetic field and surge propagation along the transmission line are both affected by losses resulting from the finite ground conductivity, which are the most significant losses.

Wire losses in TL model result from resistive losses (*I*^2^*R* losses) and the skin effect, which raises the effective resistance at higher frequencies. Ground losses are also present due to leakage currents that pass through the insulation material to the ground, depicted by the shunt conductance parameter in the TL model.

In Eqs. (11–14), losses in both the ground plane and wires are considered. The wire’s conductivity and relative permittivity are ${\sigma }_{w}$ and ${\varepsilon }_{rw}$ respectively and the ground’s conductivity and relative permittivity are ${\sigma }_{g}$ and ${\varepsilon }_{rg}$ respectively. The ground is supposed to be homogenous. The Agrawal’s coupling equations for a wire above the ground with imperfect conductivity can be expressed by Eqs. (11) and (12):

11 $$\frac{{dV_{s} (x)}}{{dx}} + Z^{\prime}I(x) = E_{x}^{e} (x,0,h)$$

12 $$\frac{{dI(x)}}{{dx}} + Y^{\prime}V_{s} (x) = 0$$

where $Z^{\prime}$ is the longitudinal impedance per-unit- length and $Y^{\prime}$ is transverse admittance per-unit- length given by:

13 $$Z^{\prime} = j\omega L^{\prime} + Z_{w} ^{\prime } + Z_{g} ^{\prime }$$

14 $$Y^{\prime} = \frac{{(G^{\prime} + j\omega C^{\prime})Y_{g} ^{\prime } }}{{G^{\prime} + C^{\prime} + Y_{g} ^{\prime } }}$$

where $G^{\prime}$ represents the longitudinal transverse conductance per unit length given by:

15 $$L^{\prime} = \frac{{\mu _{o} }}{{2\pi }}{\text{ln }}\left( {\frac{2h}{a}} \right)\;for\;h \gg a$$

16 $$C^{\prime} = \frac{{2\pi \varepsilon _{o} }}{{ln\left( {\frac{{2h}}{a}} \right)}}\;for\;h \gg a$$

17 $$G^{\prime} = \frac{{\sigma _{{air}} }}{{\varepsilon _{o} }}C^{\prime}$$

where ${Z}_{w}^{\prime}$ is the internal impedance per-unit-length of the wire, $\it{\Upsilon}_w$ is the propagation constant in the wire (*σ*_*w*_ and *ε*_*rw*_ being the wire conductivity and relative permittivity), *I*_*o*_ and *I*_*1*_ are the modified Bessel functions of zero and first order respectively.

18 $$\it{\Upsilon}_{w} = \sqrt {j\omega \mu _{o} (\sigma _{w} + j\omega \varepsilon _{o} \varepsilon _{{rw}} )}$$

19 $$Z_{w} ^{\prime } = \frac{{\Upsilon _{w} I_{o} \left( {\Upsilon _{w} a} \right)}}{{2\pi a\sigma _{w} I_{1} (\Upsilon _{w} a)}}$$

where ${Z}_{g}^{\prime}$ is the ground impedance, $\it{\Upsilon}_g$ is the propagation constant in the ground (*σ*_*g*_ and *ε*_*rg*_ being the ground conductivity and relative permittivity), ${{Y}_{g}}^{\prime}$ is the ground admittance and *h* is TL height.

20 $$\it{\Upsilon}g=\sqrt{j\omega {\mu }_{o}({\sigma }_{g}+j\omega {\varepsilon }_{o}{\varepsilon }_{rg})}$$

21 $$Z_{g} ^{\prime } = \frac{{j\omega \mu _{o} }}{{2\pi }}{\text{ln}}\left( {\frac{{1 + \Upsilon _{g} h}}{{\Upsilon _{g} h}}} \right)$$

22 $$Y_{g} ^{\prime } = \frac{{\Upsilon _{{g }} ^{2}}}{{Z_{g} ^{\prime } }}$$

Through the utilization of Green’s functions, the voltage solution stemming from a point voltage source can be determined. This methodology facilitates the resolution of the field-to-TL coupling equations and satisfies the boundary conditions.

The green’s function at a place *x*_*p*_ along the line is given by Eq. (23):

23 $$G_{v} (x;x_{p}) = \frac{{\delta e^{{ - \gamma L}} }}{{2(1 - \rho _{1} \rho _{2} e^{{ - 2\gamma L}} )}}(e^{{ - \gamma (x_{l} - L)}} + \delta \rho _{2} e^{{\gamma (x_{l} - L)}} )(e^{{\gamma x_{{sm}} }} - \delta \rho _{1} e^{{ - \gamma x_{{sm}} }} )$$

where *x*_*sm*_ represents the smaller of ${x}_{p}$ or *x*, ${x}_{l}$ represents the larger of *x*_*p*_ or *x*, *δ* = 1 for *x*_*p*_ < *x*, *δ* =  − 1 for *x*_*p*_ > *x*, *γ* represents the complex propagation constant along the TL.* ρ*_*1*_ and* ρ*_*2*_ are the voltage reflection coefficients at the loads of the TL, and *L* is length of TL.

The integrals of the Green’s functions can be used to represent the solutions involving scattered voltage by (24).

24 $$V_{s} (x) = \smallint _{0}^{L} G_{v} (x;x_{p} )E_{x} dx_{s} + G_{v}(x;0)\smallint _{0}^{h} E_{z}^{e} (0,0,z)dz - G_{v} (x;L)\smallint _{0}^{h} E_{z}^{e} (L,0,z)dz$$

25 $$V_{i} (x) = - \smallint _{0}^{h} E_{z}^{e} (x,0,z)dz$$

LIV can be calculated by adding incident voltage (*V*_*i*_) to scattered voltage (*V*_*s*_) by (26).

26 $${\text{LIV}}\left( x \right) = \, V_{s} \left( x \right) + \, V_{i} \left( x \right)$$

### Frequency-dependent models for the soil conductivity and relative permittivity

This section provides a thorough analysis of the relationship between soil electrical characteristics and the frequency of LEMP and LIV. The computation of the LEMF and the LEMF-to-line coupling equations often ignore frequency dependence of the soil electrical characteristics^14^. It is typical to assume that soil conductivity is constant and equal to the value recorded at low frequencies. Additionally, it is well known that the relative permittivity of soil varies from 4 to 81 at low frequencies^6^. while it is typically assumed to be 10 or 20 in LEMP investigations^6,17^.

This section examines the impact of frequency dependence of soil electrical characteristics on LEMP and LIV. The frequency dependence of soil conductivity and relative permittivity is caused by various factors. These factors consist of dipolar molecule polarization, the polarization of counter ions due to their diffusion (caused by the separation of cations and anions), interfacial polarization (also known as Maxwell–Wagner polarization), and various conduction and loss mechanisms. These factors depend on a specific frequency interval^6,15^. As a result, representing an accurate soil model improves the evaluation of LEMP and LIV on TL^15^. We utilized the following analytical equations proposed by Smith -Longmire model and Alipio -Visacro model in our assessment^15^.

### Smith-Longmire Model

Longmire and Longley proposed in formulation for the soil parameters using Scott’s data, which was further developed by Smith and Longmire in^6,15^. As a result, the expressions for relative permeability and ground conductivity are given by:

27 $$\varepsilon _{{rg}} \left( f \right)_{\text{Smith-Longmire Model}} = \varepsilon _{\infty } + \sum\limits_{{n = 1}}^{{14}} {\frac{{a_{n} }}{{1 + \left( {\frac{f}{{\left( {\left( {\frac{p}{{10}}} \right)^{{1.28}} *10^{{n - 1}} } \right)}}^{2} } \right)^{2} }}}$$

28 $$\sigma g(f)_{\text{Smith-Longmire Model}} = \sigma _{0} + 2\pi \varepsilon _{o} \sum\limits_{{n = 1}}^{{14}} {\frac{{a_{n} \left( {\left( {\frac{p}{{10}}} \right)^{{1.28}} *10^{{n - 1}} } \right)\left( {\frac{f}{{\left( {\left( {\frac{p}{{10}}} \right)^{{1.28}} *10^{{n - 1}} } \right)}}} \right)^{2} }}{{1 + \left( {\frac{f}{{\left( {\left( {\frac{p}{{10}}} \right)^{{1.28}} *10^{{n - 1}} } \right)}}} \right)^{2} }}}$$

$$\sigma _{o} = 8 \times 10^{{ - 3}} \left( {\frac{p}{{10}}} \right)^{{1.54}} [{\text{S}}/{\text{m}}],\varepsilon _{\infty } = 5$$

where *a*_*n*_ is a coefficient shown in Table 1 for various values of n, *σ*_*o*_ represents the low-frequency conductivity at 100 Hz, *f* represents the frequency ranging from dc to 5 MHz, ${\varepsilon }_{rg}$ (*f*) represents the relative permittivity, *σ*_*g*_ (*f*) represents soil conductivity at each frequency, and *p* is known as the percentage of moisture soil^6^.

**Table 1:** *a*_*n*_ coefficient extracted from^6^.

n	1	2	3	4	5
a_n_	3.4 × 10^6^	2.74 × 10^5^	2.58 × 10^4^	3.38 × 10^3^	5.26 × 10^2^
n	6	7	8	9	10
a_n_	1.33 × 10^2^	2.72 × 10	1.25 × 10	4.8	2.17
n	11	12	13	14	
a_n_	0.98	0.392	0.173	0	

### Alipio-Visacro Model

Another soil model was proposed by Visacro and Alipio^15^. They represent the soil characteristics’ frequency dependency by the following Eqs. (29) and (30).

29 $$\sigma _{g} (f)_{\textbf{Alipio-Visacro Model}} = \left[ {\sigma _{o} + \sigma _{o} h(\sigma _{o} )\left( {\frac{f}{{10^{6} }}} \right)^{\gamma } } \right] \times 10^{{ - 3}}$$

30 $$\varepsilon _{{rg}} \left( f \right)_{\textbf{Alipio-Visacro Model}} = \frac{{\varepsilon _{\infty } ^{\prime } }}{{\varepsilon _{o} }} + \frac{{{\text{tan}}\left( {\frac{{\pi \gamma }}{2}} \right) \cdot 10^{{ - 3}} }}{{2\pi \varepsilon _{o} \left( {10^{6} } \right)^{\gamma } }}\sigma _{o} h(\sigma _{o} )f^{{\gamma - 1}}$$

where *σ*_100Hz_ is the value of *σ*_*o*_ [mS/m],* h*(*σ*_*o*_) = 1.26 × ${\sigma }_{o}^{-0.73}$ and $\gamma$ = 0.54.

### Comparison between frequency-dependent and independent models

**Fig. 5:**
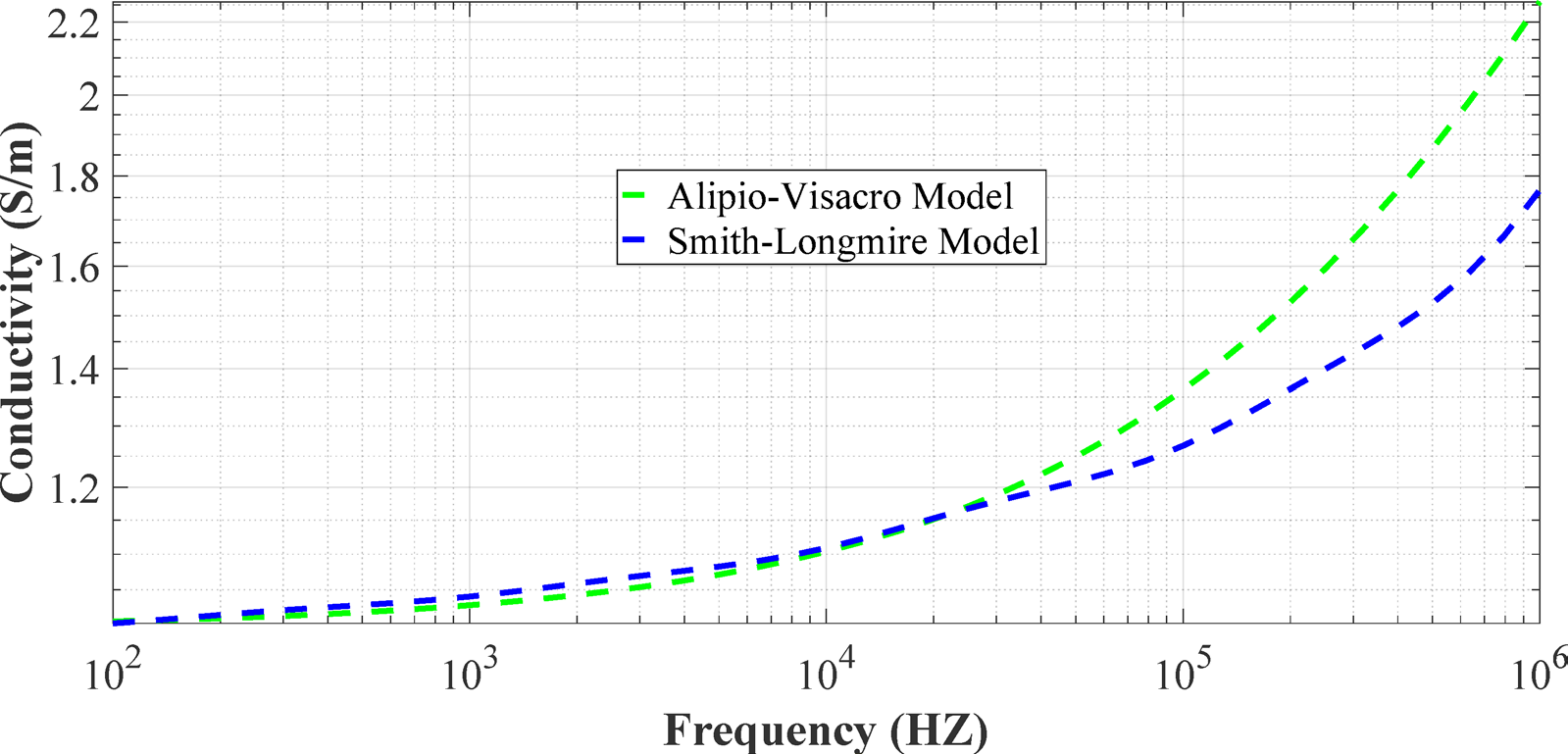
The conductivity of two frequency-dependent models at *p* = 2.6%, and ${\varepsilon }_{rg}$ = 10 is presented as a function of frequency.

**Fig. 6:**
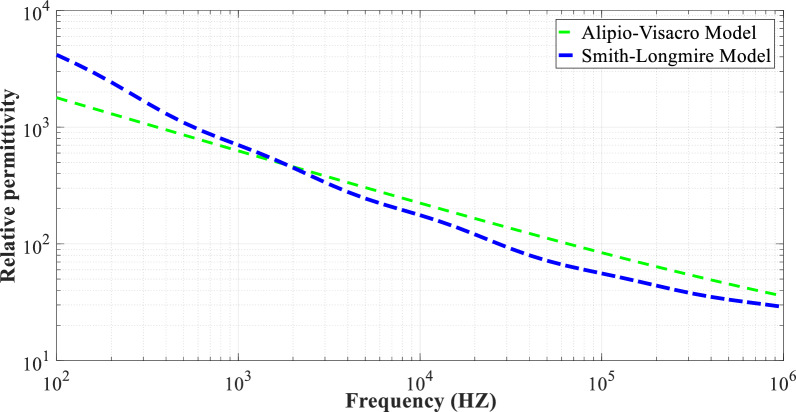
The relative permittivity of two frequency-dependent soil models at *p* = 2.6%, and ${\varepsilon }_{rg}$ = 10 is presented as a function of frequency.

Figures 5 and 6 show the soil conductivity and soil relative permittivity variation over frequency for two frequency-dependent soil models (Alipio-Visacro model and Smith-Longmire model), respectively.

**Fig. 7:**
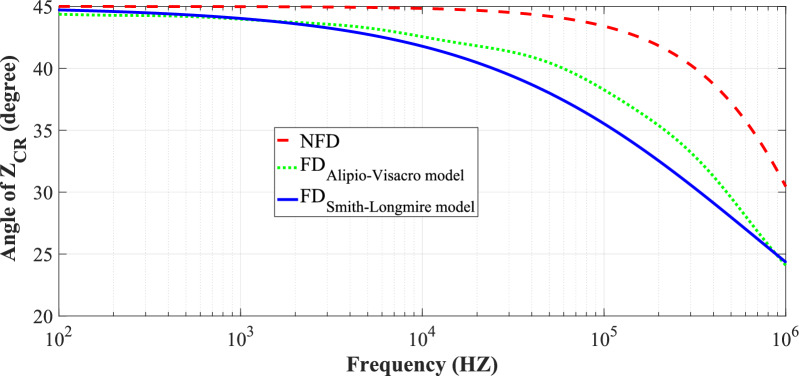
The variation of *Z*_*CR*_ angle variation with frequency for three soil models at *p* = 2.6%, ${\varepsilon }_{rg}$ = 10, *σ*_*o*_ = 0.001 S/m.

**Fig. 8:**
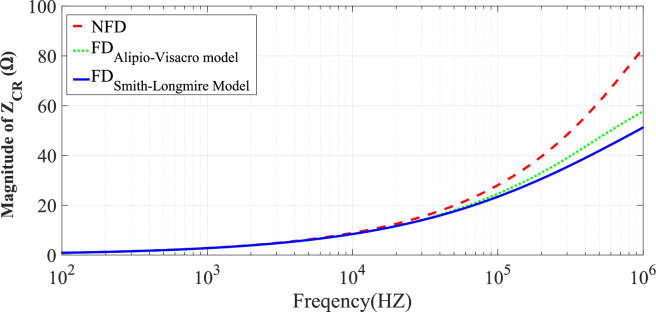
The variation of *Z*_*CR*_ magnitude with frequency for three soil models at *p* = 2.6%, ${\varepsilon }_{rg}$= 10, *σ*_*o*_ = 0.001 S/m.

Figures 7 and 8 illustrate the variation in the angle and magnitude of *Z*_*CR*_ in the CR formula with frequency for cases: non-frequency-dependent (NFD) model (fixed soil parameters as ${\varepsilon }_{rg}$ and *σ*_*o*_), Smith-Longmire and Alipio-Visacro frequency-dependent (FD) models (FD soil parameters ${\varepsilon }_{rg}$ (*f*) and* σ*_*g*_ (*f*)).

## Result

The effects of ground losses ${Z}_{g}^{\prime}$ and wire losses ${Z}_{w }^{\prime}$ in TL model on LIV are studied by considering both NFD model and two FD models of soil parameters. This study was conducted by varying *v* (40 m/*µ*s, 120 m/*µ*s, and 200 m/*µ*s respectively), *h* (6 m and 10 m respectively), and *d* (50 m and 100 m respectively). LIV values are computed at two points along the TL: the midpoint and 500 m from the midpoint. The midpoint refers to the position on the TL directly in front of the lightning channel. The results are compared against the FDTD method, as referred in Appendix A (uploaded in supplementary material, labelled “supplementary information-Appendix”).

### Effect of ground losses in the TL model on LIV with various cases of lightning velocity

**Fig. 9:**
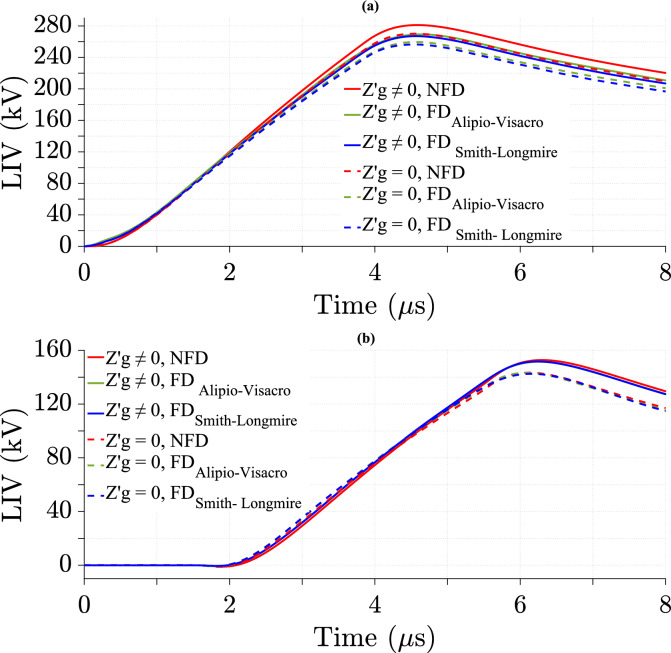
LIV values using three soil models using CR formula in frequency domain at *v* = 40 m/*µ*s for *σ*_*o*_ = 0.001 S/m, *σ*_*g*_ (*f*) at *p* = 2.6%, *h* = 10 m, *d* = 50 m, and ${\varepsilon }_{rg}$ = 10. (**a**) At the midpoint; (**b**) at 500 m from the midpoint.

**Fig. 10:**
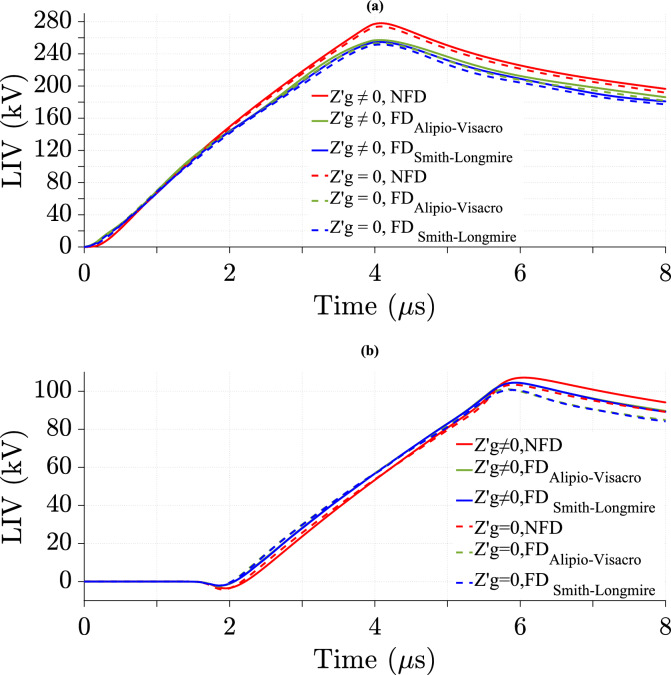
LIV values using three soil models using CR formula in frequency domain at *v* = 120 m/*µ*s for *σ*_*o*_ = 0.001 S/m, *σ*_*g*_ (*f*) at *p* = 2.6%, *h* = 10 m, *d* = 50 m, and ${\varepsilon }_{rg}$ = 10. (**a**) at the midpoint; (**b**) at 500 m from the midpoint.

**Fig. 11:**
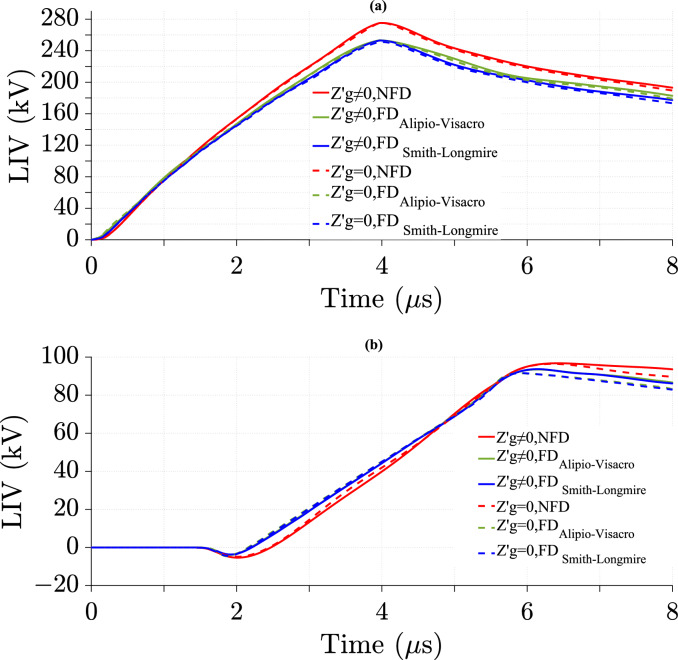
LIV values using three soil models using CR formula in frequency domain at *v* = 200 m/*µ*s for *σ*_*o*_ = 0.001 S/m, *σ*_*g*_ (*f*) at *p* = 2.6%, *h* = 10 m, *d* = 50 m, and ${\varepsilon }_{rg}$ = 10. (**a**) at the midpoint; (**b**) at 500 m from the midpoint.

The impact of ground losses in the TL model on LIV is studied using the NFD model and two FD models at different values for *v*. Figures 9, 10, and 11 show the variation of LIV over time for *v* equal to 40 m/*µ*s, 120 m/*µ*s, and 200 m/*µ*s, respectively. As shown in the Figures that the LIV is highest in value at midpoint with NFD soil model while the impact of the soil model diminishes at 500 m from midpoint.

The percentage of the difference in LIV peak values with and without considering the ground losses are computed using Eq. (31) to obviously show the effect of ground losses on LIVs.

31 $$\Delta {\text{LIV}}_{{{\text{effect}}\;{\text{of}}\;{\text{ground}}\;{\text{losses}}}} {\text{ }}(\% ) = \frac{{{\text{LIV}}_{{{\text{with}}\;{\text{ground}}\;{\text{losses }}}} - {\text{LIV}}_{{{\text{without}}\;{\text{ground}}\;{\text{losses}}}} }}{{{\text{LIV}}_{{{\text{with}}\;{\text{ground}}\;{\text{losses }}}} }} \times 100$$

where ${\text{LIV}}_{\text{with ground losses}}$ represents the peak value of LIV considering ground losses, and the peak value of LIV ignoring ground losses is represented by ${\text{LIV}}_{\text{without ground losses}}$.

The results show the effect of ground losses in the TL model on LIV by comparing cases with and without considering them at different *v*. The data is analyzed for *v* of 40 m/*µ*s, 120 m/*µ*s and 200 m/*µ*s at two positions along the TL: the midpoint, as shown in Table 2, and 500 m from the midpoint, as shown in Table 3. This study shows that at lower *v* (40 m/*µ*s and 120 m/*µ*s), the difference in LIV between the two cases is more significant, whereas at higher *v* (200 m/*µ*s), the difference percentage in LIV peak values decreases considerably. Additionally, FD soil models, including the Alipio-Visacro and Smith-Longmire models, exhibit greater LIV peak value differences compared to the NFD model at higher *v* (200 m/*µ*s). However, the Alipio-Visacro model produces lower LIV peak value differences compared to the NFD model and the Smith-Longmire model at *v* of 40 and 120 m/*µ*s. Furthermore, the LIV peak value difference is consistently higher at 500m from the midpoint than at the midpoint itself. For instance, at 40 m/*µ*s, the LIV difference increases from 4.07% at the midpoint to 6.5% at 500m for the NFD case, following a similar trend in FD models. The results indicate that including ground losses increase LIV peak value, as shown by the positive percentage of difference. Moreover, higher *v* reduces the impact of ground losses on LIV. These findings highlight the necessity of accounting for ground losses in LIV calculations, particularly at lower *v* and farther along the TL.

**Table 2:** Percentage of difference in LIV peak values at the midpoint of the TL using three soil models at different *v*, as calculated from Eq. (31).

Velocity (m/*µ*s)	Soil model			
	NFD (%)	FD_Alipio-Visacro Model_ (%)	FD_Smith-Longmire Model_ (%)	Figures
40	4.07	3.7	4.1	9a
120	1.5	1.2	1.5	10a
200	0.01	0.78	0.68	11a

**Table 3:** Percentage of difference in LIV peak values at 500 m from the midpoint of the TL using three soil models at different *v*, as calculated from Eq. (31).

Velocity (m/*µ*s)	Soil model			
	NFD (%)	FD_Alipio-Visacro Model_	FD_Smith-Longmire Model_	Figures
40	6.5	5.8	6.5	9b
120	3.6	3.34	3.82	10b
200	0.35	2.13	2.3	11b

The impact of soil model on LIV magnitude was evaluated by comparing peak values from the NFD and FD models (Alipio-Visacro model and Smith-Longmire model) using the Eq. (32):

32 $$\Delta {\text{LIV}}_{{{\text{NFD}} \text{-} {\text{FD}}}} {\text{ }}(\% ) = \frac{{{\text{LIV}}_{{{\text{using}}\;{\text{NFD}}\;{\text{model }}}} - {\text{LIV}}_{{{\text{using}}\;{\text{FD}}\;{\text{model}}}} }}{{{\text{LIV}}_{{{\text{using}}\;{\text{NFD}}\;{\text{model }}}} }} \times 100$$

where ${\text{LIV}}_{\text{using NFD model}}$ represents the peak LIV from the NFD model, and ${\text{LIV}}_{\text{using FD model}}$ represents the peak LIV from the FD model.

Tables 4 and 5 show that LIV peak value is lower with FD soil models. While the difference in LIV peak value between NFD and FD models is larger at higher *v*.

**Table 4:** Percentage of difference in peak values of LIV with ground losses between the NFD and FD models at different *v*, as calculated from Eq. (32).

Velocity (m/*µ*s)	Soil model			
	At midpoint of TL		At 500 m from the midpoint of TL	
	NFD_FD_Aipio-Visacro Model_ (%)	NFD_FD_Smith-Longmire Model_ (%)	NFD_FD_Alipio-Visacro Model_ (%)	NFD_FD_Smith-Longmire Model_
40	4.2	5	0.82	0.7
120	7.5	8.4	2.5	2.47
200	7.9	8.5	3.3	3.2

**Table 5:** Percentage of difference in peak values of LIV without ground losses between the NFD and FD models at different *v*, as calculated from Eq. (32).

Velocity (m/*µ*s)	Soil model			
	At midpoint of TL		At 500 m from the midpoint of TL	
	NFD_FD_Alipio-Visacro Model_ (%)	NFD_FD_Smith-Longmire Model_ (%)	NFD_FD_Alipio-Visacro Model_ (%)	NFD_FD_Smith-Longmire Model_ (%)
40	3.9	5	0.13	0.6
120	7.5	8.2	2.24	2.65
200	8.7	8.7	5	5

In Comparison to the NFD model, the Smith-Longmire model predicts a slightly higher percentage of difference in LIV with ground losses than the Alipio-Visacro model at the midpoint of the TL, as shown in Table 4. However, at 500 m from the midpoint, the opposite occurs, with the Alipio-Visacro model compared to the NFD model predicting a slightly higher percentage of difference in LIV with ground losses than the Smith-Longmire model, as shown in Table 4. Table 5 shows that the Smith-Longmire model estimates the percentage of difference in LIV without ground losses to be equal to or slightly higher than that of the Alipio-Visacro model compared to the NFD model. These findings highlight the importance of using FD soil models in LIV calculations especially at higher *v*.

### Effect of ground losses in the TL model on LIV with various cases of transmission line height

**Fig. 12:**
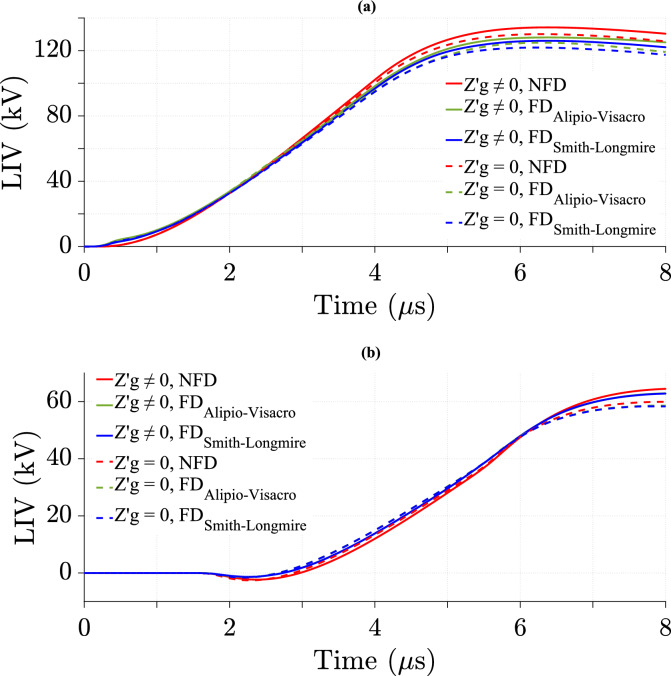
LIV results using three ground models with CR formula in frequency domain at *h* = 6 m, and *d* = 100 m for *σ*_*o*_ = 0.001 S/m, *σ*_*g*_ (*f* ) at *p* = 2.6%, *v* = 40 m/*µ*s, and ${\varepsilon }_{rg}$ = 10. (**a**) at the midpoint; (**b**) at 500 m from the midpoint.

**Fig. 13:**
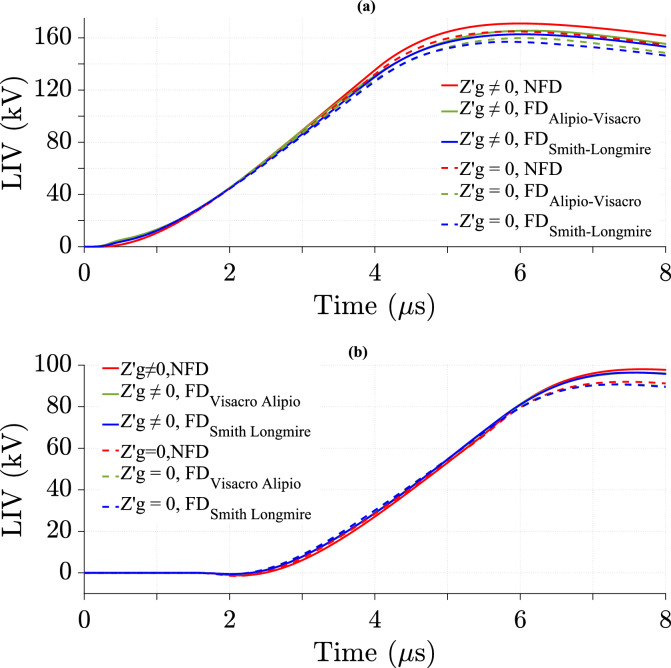
LIV results using three ground models with CR formula in frequency domain at *h* = 10 m for *σ*_*o*_ = 0.001 S/m, *σ*_*g*_ (*f*) at *p* = 2.6%, *v* = 40 m/*µ*s, ${\varepsilon }_{rg}$ = 10, and *d* = 100 m. (**a**) at the midpoint; (**b**) at 500 m from the midpoint.

This study presents the impact of ground losses in the TL model on LIV using the NFD model and two FD soil models at different *h*. Figures 12 and 13 show the LIV variation over time for *h* = 6 m and *h* = 10 m, respectively.

At the midpoint of the TL, the percentage of difference in LIV peak value increases with increasing *h*, as shown in Table 6, indicating a greater impact of ground losses at higher elevations. In contrast, at 500 m, the difference decreases with increasing *h*, as illustrated in Table 7, providing weaker influence at greater distances. The ground losses have stronger impact at 500 m from the midpoint than at midpoint, with higher percentage of differences in LIV peak value across all *h* values. Smith-Longmire shows the highest impact of ground losses at *h* = 6 m. At *h* = 10 m, NFD has a similar or higher impact than the Smith-Longmire models. The Alipio-Visacro model consistently shows the lowest influence of ground losses.

**Table 6:** Percentage of difference in LIV peak values at the midpoint of the TL using three soil models at different *h*, as calculated from Eq. (31).

Height (m)	Soil model			
	NFD (%)	FD_Alipio-Visacro Model_ (%)	FD_Smith-Longmire Model_ (%)	Figures
6	3.2	3	3.4	12a
10	3.7	3.5	3.7	13a

**Table 7:** Percentage of difference in LIV peak values at 500m from the midpoint of the TL using three soil models at different *h*, as calculated from Eq. (31).

Height (m)	Soil model			
	NFD (%)	FD_Alipio-Visacro Model_ (%)	FD_Smith-Longmire Model_ (%)	Figures
6	7.6	7.3	7.7	12b
10	6.6	6.1	6.3	13b

Tables 8 and 9 show that the difference in peak LIV values between the NFD and FD models is larger at lower *h* with and without considering the ground losses. This difference is noticeable at both locations, with greater values at the midpoint of the TL. Compared to the NFD model, the Smith-Longmire model shows a larger difference in peak LIV values than the Alipio-Visacro model.

**Table 8:** Percentage of difference in peak values of LIV with ground losses between the NFD and FD models at different *h*, as calculated from Eq. (32).

Height (m)	Soil model			
	At midpoint of TL		At 500 m from the midpoint of TL	
	NFD_FD_Alipio- Visacro Model_ (%)	NFD_FD_Smith-Longmire Model_ (%)	NFD_FD_Alipio-Visacro Model_ (%)	NFD_FD_Smith-Longmire Model_ (%)
6	4.6	6.1	2.5	2.6
10	3.2	4.8	1.7	1.8

**Table 9:** Percentage of difference in peak values of LIV without ground losses between the NFD and FD models at different *h*, as calculated from Eq. (32).

Height (m)	Soil model			
	At midpoint of TL		At 500 m from the midpoint of TL	
	NFD_FD_Alipio- Visacro Model_ (%)	NFD_FD_Smith-Longmire Model_ (%)	NFD_FD_Alipio-Visacro Model_ (%)	NFD_FD_Smith-Longmire Model_ (%)
6	4	6.3	2.2	2.7
10	3	4.8	1.25	1.4

### Effect of ground losses in the TL model on LIV with various cases of distance between transmission line and lightning strike

**Fig. 14:**
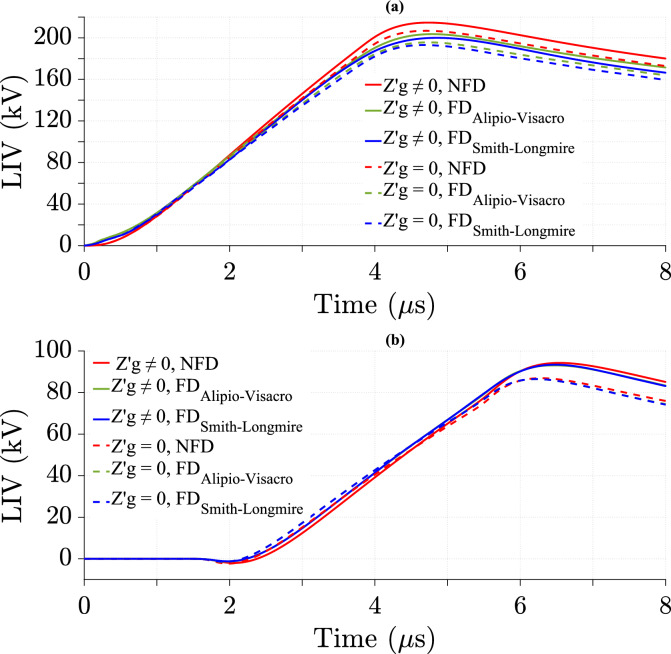
LIV results using three ground models with CR formula in frequency domain at *d* = 50 m for *σ*_*o*_ = 0.001 S/m, *σ*_*g*_ (*f*) at *p* = 2.6%, *v* = 40 m/*µ*s, ${\varepsilon }_{rg}$= 10, and *h* = 6 m. (**a**) at the midpoint; (**b**) at 500 m from the midpoint.

The effect of ground losses in the TL model on LIV is analyzed using the NFD model and two FD models for different values of *d*. Figures 12 and 14 illustrate the variation of LIV over time for *d* = 100 m and *d* = 50 m, respectively.

The results show the impact of ground losses on the LIV peak values at different *d*. Tables 10 and 11 show that as *d* increases from 50 to 100 m, the LIV peak difference decreases. This indicates a stronger impact of ground losses when the strike is closer to the TL. Additionally, the LIV peak value difference is greater at 500 m from the midpoint than at the midpoint. Considering ground losses increases the LIV peak value, with positive percentage of differences in Tables 10 and 11. At 500 m from the TL midpoint, the NFD model has the highest percentage of difference at 50 m, as shown in Table 11. However, at 100 m, the Smith-Longmire model slightly exceeds the NFD model, as described in Table 11. At the TL midpoint, the Alipio-Visacro model shows a slightly higher percentage of difference than both the NFD and Smith-Longmire models at 50 m, as illustrated in Table 10. However, at 100 m, the Alipio-Visacro model produces the lowest percentage value, as shown in Table 10.

**Table 10:** Percentage of difference in LIV peak values at the midpoint of the TL using three soil models at different *d*, as calculated from Eq. (31).

Distance (m)	Soil model			
	NFD (%)	FD_Alipio-Visacro Model_ (%)	FD_Smith-Longmire Model_ (%)	Figures
50	3.8	4	3.6	14a
100	3.2	3	3.4	12a

**Table 11:** Percentage of difference in LIV peak values at 500 m from the midpoint of the TL using three soil models at different *d*, as calculated from Eq. (31).

Distance (m)	Soil model			
	NFD (%)	FD_Alipio-Visacro Model_ (%)	FD_Smith-Longmire Model_ (%)	Figures
50	8.6	7.7	8	14b
100	7.6	7.3	7.7	12b

Tables 12 and 13 compare the percentage of differences in LIV peak values between the NFD and FD models at varying *d*, with and without ground losses, respectively. The results show that LIV peak value differences are generally higher at the TL midpoint than at 500 m from the midpoint. The NFD model produces higher LIV peak values than both FD models. The difference between the Smith-Longmire and NFD models is larger than that between the Alipio-Visacro and NFD models, especially at the midpoint. However, at 500 m from the midpoint, the Alipio-Visacro model shows a slightly higher difference from the NFD model at 50 m. As *d* increases from 50 to 100 m, the LIV difference decreases at the midpoint but increases at 500 m from the midpoint. These findings highlight the significant role of the frequency-dependent soil model in LIV calculations.

**Table 12:** Percentage of difference in peak values of LIV with ground losses between the NFD and FD models at different *d*, as calculated from Eq. (32).

Distance (m)	Soil model			
	At midpoint of TL		At 500 m from the midpoint of TL	
	NFD_FD_Alipio- Visacro Model_ (%)	NFD_FD_Smith-Longmire Model_ (%)	NFD_FD_Alipio-Visacro Model_ (%)	NFD_FD_Smith-Longmire Model_ (%)
50	5.2	6.8	1.3	1
100	4.6	6.1	2.5	2.6

**Table 13:** Percentage of difference in peak values of LIV without ground losses between the NFD and FD models at different *d*, as calculated from Eq. (32).

Distance (m)	Soil model			
	At midpoint of TL		At 500 m from the midpoint of TL	
	NFD_FD_Alipio-Visacro Model_ (%)	NFD_FD_Smith-Longmire Model_ (%)	NFD_FD_Alipio-Visacro Model_ (%)	NFD_FD_Smith-Longmire Model_ (%)
50	5.4	6.6	0.5	0.47
100	4	6.3	2.2	2.7

### Effect of wire losses in the TL model on LIV

**Fig. 15:**
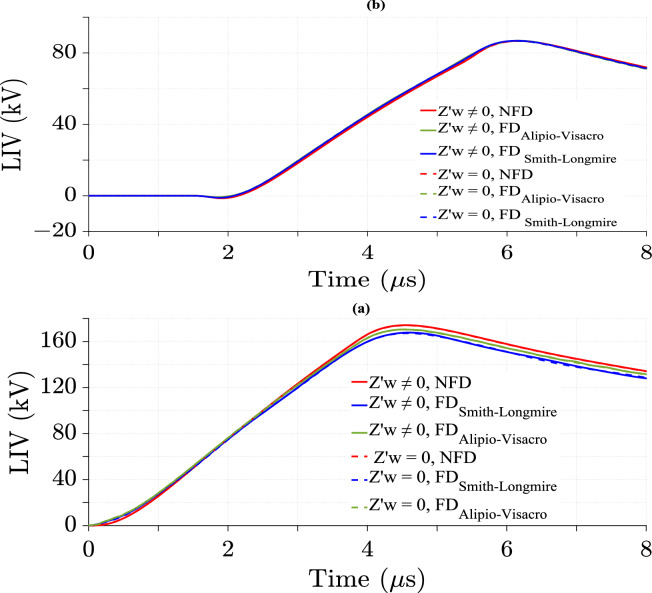
LIV results using three ground models with CR formula in frequency domain at *d* = 50 m, *σ*_*w*_ = 58 MS/m, *v* = 40 m/*µ*s, *h* = 6 m, ${\varepsilon }_{rw}$ = 0.994. (**a**) At the midpoint; (**b**) at 500 m from the midpoint.

The impact of wire losses in the TL model is analyzed using the NFD model and two FD models. Figure 15 illustrates the LIV variation over time with and without wire losses.

Figure 15 shows no noticeable difference in LIV values between considering and ignoring wire losses for the three soil models. Therefore, wire losses have a negligible effect in this study.

## Discussion

**Fig. 16:**
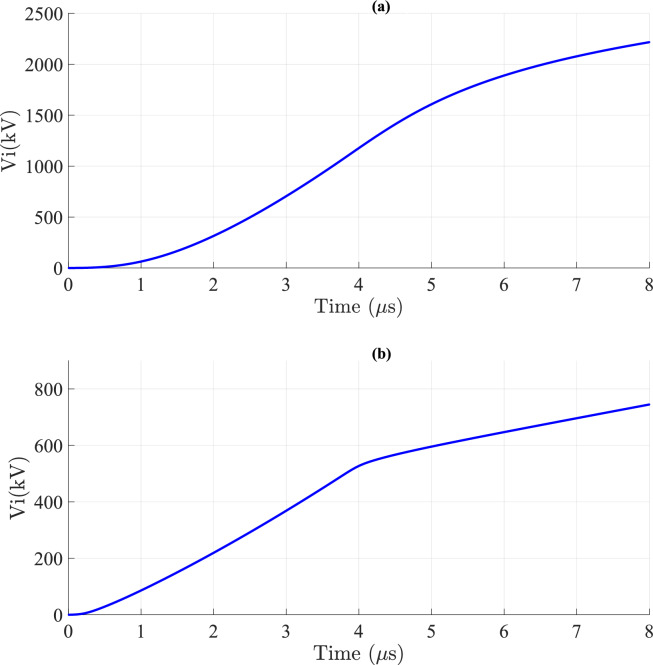
*V*_*i*_ result at the midpoint of TL for *σ*_*o*_ = 0.001 S/m, *h* = 10 m, *d* = 50 m, and ${\varepsilon }_{rg}$= 10. (**a**) at *v* = 40 m/*µ*s. (**b**) at *v* = 200 m/*µ*s.

In this section, the impact of ground losses on LIV is analyzed at varying *v*. As outlined in the adopted methodology, LIV comprises two components: *V*_*s*_ and *V*_*i*_ as referenced in Eq. (26). Ground losses affect *V*_*s*_ while *V*_*i*_ remains unaffected. The waveform of *V*_*i*_ is consistently unipolar, appearing in the positive portion, as illustrated in Fig. 16. This figure demonstrates that *v* significantly influences the magnitude of *V*_*i*_ with *V*_*i*_ being considerably higher for *v* = 40 m/*µ*s compared to *v* = 200 m/*µ*s.

**Fig. 17:**
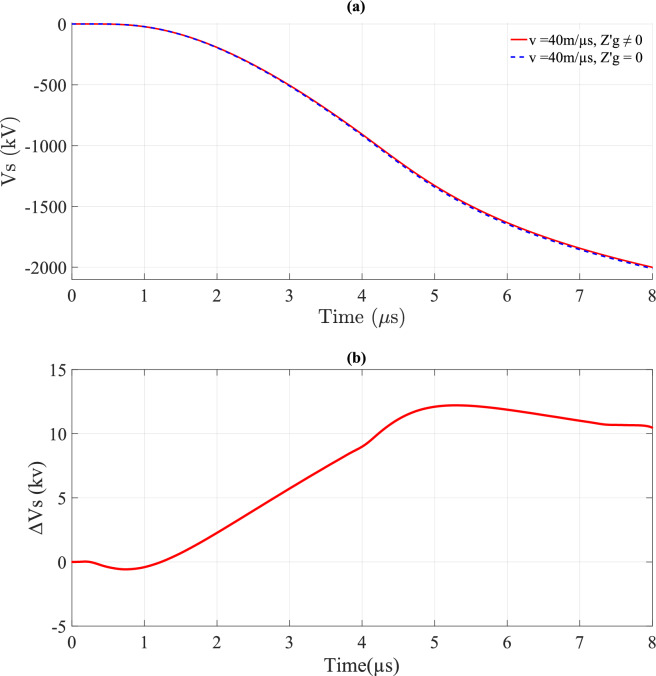
*V*_*s*_ result for *v* = 40 m/*µ*s at the midpoint of TL with *σ*_*o*_ = 0.001 S/m, *h* = 10 m, *d* = 50 m, and ${\varepsilon }_{rg}$ = 10. (**a**) *V*_*s*_; (**b**) ∆*V*_*s*_ difference between considering and ignoring ground losses.

**Fig. 18:**
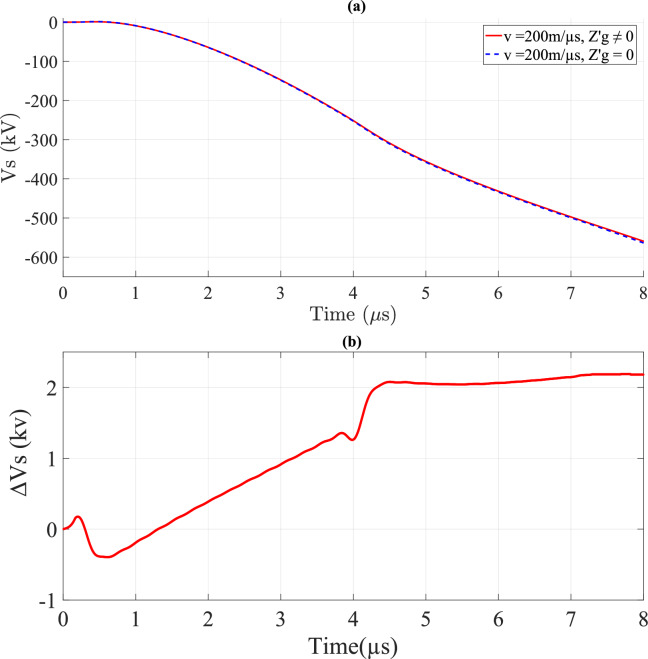
*V*_*s*_ result for *v* = 200 m/*µ*s at the midpoint of TL with *σ*_*o*_ = 0.001 S/m, *h* = 10 m, *d* = 50 m, and ${\varepsilon }_{rg}$ = 10. (**a**) *V*_*s*_; (**b**) ∆*V*_*s*_ difference between considering and ignoring ground losses.

Figures 17 and 18 depict *V*_*s*_ and ∆*V*_*s*_ with and without ground losses, respectively. From Figs. 17a and 18a, it is evident that the waveform of *V*_*s*_ is also unipolar but appears in the negative portion. These figures further reveal that *v* has a significant impact on *V*_*s*_, similar to its effect on *V*_*i*_. Additionally, Figs. 17b and 18b illustrate that the variation of ∆*V*_*s*_ over time becomes negligible for *v* = 200 m/*µ*s, unlike the case for *v* = 40 m/*µ*s, where the variation is more pronounced.

This phenomenon is attributed to the higher magnitudes of the HEF and VEF at lower *v* compared to higher values of *v* at short *d*, as discussed in ^30^. Consequently, the magnitudes of the induced current *I*(*x*) through the wire of the TL increase at lower *v*, making the characteristics of the TL more pronounced. As a result, the LIV magnitudes with ground losses are higher than those without ground losses, as illustrated in Fig. 9. In the case of higher *v*, the values of *I*(*x*) decrease through the wire of the TL, making the characteristics of the TL less noticeable. As a result, the effect of ground losses becomes negligible at higher *v*, as shown in Fig. 11.

This study investigates the impact of using FD model of the soil on the LIV. Three soil models are simultaneously used to represent ground losses in TL. The results illustrate that ground losses significantly influence LIV, particularly in relation to *v*. An accurate estimation of LIVs is crucial for the studies of insulation coordination, and lightning protection to improve the performance of medium and low voltage overhead lines with indirect strokes. From an engineering perspective, this work provides valuable insights for designing more resilient transmission networks, ensuring safer operation, and reducing the economic impact of lightning-related failures.

## Conclusion

This paper evaluates lightning-induced voltage values with a specific emphasis on accounting for ground losses in transmission line model. The ground losses are represented using three soil models: non-frequency-dependent model and two frequency-dependent models.

The key findings are as follows:

1. Considering ground losses increase lightning-induced voltage magnitude compared to ignoring them.
2. The increase in lightning-induced voltages when considering the ground losses in transmission line model becomes more significant with lower values of lightning velocity at any point on transmission line.
3. The increase in lightning-induced voltages due to considering ground losses in transmission line model becomes more significant as the value of transmission line height decreases with moving away of midpoint of transmission line.
4. As the height of the transmission line increases at its midpoint, the effect of ground losses on lightning-induced voltages becomes more pronounced.
5. The percentage of increase in peak value of lightning-induced voltage becomes more significant for distance between lightning strike and transmission line = 50 m compered to distance = 100 m at any point of transmission line.
6. The increase in lightning-induced voltage magnitudes using the non-frequency-dependent model is more than that increase in lightning-induced voltage magnitudes using the two frequency-dependent models.
7. Wire losses do not affect the computed lightning-induced voltage.

## Supplementary Information

Supplementary Information 1.

Supplementary Information 2.

## Data Availability

All data generated or analyzed during this study are included in this published article and its supplementary information file.
