# Supplementary material for: The impact of ground losses on estimations of lightning-induced voltage with dispersive soil parameters
Source: Sci Rep. 2025 Jul 6;15:24146. doi: 10.1038/s41598-025-08379-3 (PMC12230118; doi:10.1038/s41598-025-08379-3)
Supplement: Supplementary file 1 — Supplementary Information 1. [file 41598_2025_8379_MOESM1_ESM.zip › data/Response To Inquire .pdf]

Response To Inquire

Mathematical Models of Manuscript

1 Computation of Lightning Electromagnetic Fields

Firstly, the trapezoidal waveform is used to represent lightning strike current.

$$I(j\omega)=\frac{I_O}{T(j\omega)^2}-\frac{I_O}{T(j\omega)^2}e^{-j\omega T} \quad (1)$$

Where I_O is the peak value of lightning stroke current, and T is the rise time of current waveform.

Used data in eq(1): T = 3.8μs and I_o = 30 kA

Secondly, electromagnetic field equations are represented in perfectly conducting ground.

$$E_z(r, z, j\omega) = \frac{1}{4\pi\varepsilon_o}\Big(\int_{-H}^{H}\frac{2(z-z')^2-r^2}{R^5}\frac{1}{j\omega}I(z',j\omega) e^{-\frac{j\omega R}{c}}dz' + \int_{-H}^{H}\frac{2(z-z')^2-r^2}{cR^4}I(z',j\omega)e^{-\frac{j\omega R}{c}}dz' - \int_{-H}^{H}\frac{r^2}{c^2R^3}j\omega\, I(z',j\omega)\,e^{-\frac{j\omega R}{c}}dz'\Big) \quad (2)$$

$$H_\phi(r, z, j\omega) = \frac{1}{4\pi}\Big(\int_{-H}^{H}\frac{r}{R^3}I(z',j\omega)e^{-\frac{j\omega R}{c}}dz' + \int_{-H}^{H}\frac{r}{cR^2}j\omega\, I(z',j\omega)e^{-\frac{j\omega R}{c}}dz'\Big) \quad (3)$$

Where E_z is the vertical electric field, $H\phi$ is magnetic field intensity, $R = \sqrt{r^2-(z-z')^2}$ represents the distance between the lightning channel and the location where the field is being calculated. $I(r,j\omega)$ represent the current distribution along the lightning channel in the frequency domain. The dipole height above the ground surface is represented by h that equals to 6 m or 10m in this research, while the height of the lightning channel is represented by H, which is approximately 2500 meters above the ground surface in this investigation .the speed of light is represented as c. The horizontal distance between the lightning channel and the location where the electromagnetic field is calculated is represented as r. Additionally, the height of the point where the field is being calculated above the ground is denoted as z.

$$E_{rp}(r, z, j\omega) = \frac{1}{4\pi\varepsilon_o}\Big(\int_{-H}^{H}\frac{3r(z-z')}{R^5}\frac{1}{J\omega}I(z',j\omega)e^{-\frac{j\omega R}{C}}dz' + \int_{-H}^{H}\frac{3r(z-z')}{cR^4}I(z',j\omega)e^{\frac{j\omega R}{C}}dz' + \int_{-H}^{H}\frac{r(z-z')}{c^2R^3}j\omega e^{-\frac{j\omega R}{c}}dz' \quad (4)$$

$E_{rp}(r,z,j\omega)$ represents the horizontal electric field induced by the lightning channel at a height z and distance r, assuming the ground to be a perfect conductor.

Thirdly, horizontal electric field is only component that is influenced by lossy ground. The Cooray-Rubinstein formula is used in this work to calculate the HEF at ground level.

$$E_r(r, z, j\omega) = E_{rp}(r, z, j\omega) - H\phi(r,0,j\omega)\frac{c\mu_o}{\sqrt{\varepsilon_{rg}+\frac{\sigma_g}{j\omega\varepsilon_o}}} \quad (5)$$

Where σ_g is soil conductivity, $\varepsilon_{rg}$is soil permittivity that and ε_o is free space permittivity.

3 Field to Line Coupling Equation

Agrawal's Field-to-TL equations are shown below assuming the TL approximation applied.

$$\frac{dv^s(x)}{dx} + j\omega L' I(x) = E_x^e(x, 0, h) \quad (7)$$

$$\frac{dI(x)}{dx} + j\omega\ C' V^S(x) = 0 \quad (8)$$

Where L' is known as the longitudinal inductance per-unit-length, C' is known as transverse capacitance per unit length, I(x) is symbol of induced current through the wire, and V^s is scattered voltage.

For termination impedances Z_A and Z_B shown, the boundary condition in terms of the scattered voltage and total current.

$$V^s(0) = -Z_A\, I\ (0) + \int_0^h E_z^e(0,0,\ z)\ dz \quad (9)$$

$$V^s(L) = Z_B\, I(L) + \int_0^h E_z^e(L,0,\ z)\ dz \quad (10)$$

When calculating LIVs, losses in the wire and ground can be considered in theory. The electromagnetic field and surge propagation along the transmission line are both affected by losses resulting from the finite ground conductivity, which are the most significant losses.

In (11-14), losses in both the ground plane and wires are considered. The wire's conductivity and relative permittivity are σ_w and ε_{rw} respectively and the ground's conductivity and relative permittivity are σ_g and ε_{rg} respectively. The ground is supposed to be homogenous. The Agrawal's coupling equations for a wire above the ground with imperfect conductivity can be expressed by (11) and (12) :

$$\frac{dv^s(x)}{dx} + Z'\ I(x) = E_x^e(x,0,\ h) \quad (11)$$

$$\frac{dI(x)}{dx} + Y'\ V^S(x) = 0 \quad (12)$$

Where Z' is the longitudinal impedance per-unit- length and Y' is transverse admittance per-unit- length given by :

$$Z' = j\omega L' + Z'_w + Z'_g \quad (13)$$

$$Y' = \frac{(G' + jw\ C')Y_g'}{G' + C' + Y_g'} \quad (14)$$

Where Z' represents the longitudinal impedance per unit length and Y' represents the transverse admittance per unit length given by :

$$L' = \frac{\mu_O}{2\pi} \ln\left(\frac{2h}{a}\right)\ for\ h \gg a \quad (15)$$

$$C' = \frac{2\pi\varepsilon_O}{ln\,(\frac{2h}{a})}\ for\ h \gg a \tag{16}$$

$$G' = \frac{\sigma_{air}}{\varepsilon_o} C' \tag{17}$$

Where Z'_w is the internal impedance per-unit-length of the wire, γ_w is the propagation constant in the wire (σ_w and ε_{rw} being the wire conductivity and relative permittivity), I_o and I_1 are the modified Bessel functions of zero and first order respectively.

$$\gamma_w = \sqrt{j\omega\mu_o(\sigma_w + jw\varepsilon_o\varepsilon_{rw})} \tag{18}$$

$$Z'_w = \frac{\Upsilon_{w I_O}(\Upsilon_w a)}{2\pi a\sigma_{w I_1}(\Upsilon_w a)} \tag{19}$$

Where Z'_g is the ground impedance, γ_g is the propagation constant in the ground (σ_g and ε_{rg} being the ground conductivity and relative permittivity) and Y'_g is the ground admittance.

$$\gamma_g = \sqrt{j\omega\mu_o(\sigma_g + j\omega\varepsilon_o\varepsilon_{rg})} \tag{20}$$

$$Z'_g = \frac{j\omega\mu_o}{2\pi} \ln\,(\frac{1+\Upsilon_g h}{\Upsilon_g h}) \tag{21}$$

$$Y'_g = \frac{\Upsilon_g^2}{Z'_g} \tag{22}$$

Through the utilization of Green's functions, the voltage solution stemming from a point voltage source can be determined. This methodology facilitates the resolution of the field-to-transmission-line coupling equations and satisfies the boundary conditions.

The green's function at a place x_p along the line is given by (23) :

$$G_V(x;\, x_p) = \frac{\delta e^{-\gamma L}}{2(1-\rho_1\,\rho_2 e^{-2\gamma L})}\,(e^{-\gamma\,(x_l - L)} + \delta\rho_2\, e^{\,\gamma\,(x_l - L)})$$

$$(e^{\,\gamma\, x_{sm}} - \delta\rho_1\, e^{-\gamma\, x_{sm}}) \tag{23}$$

Where x_{sm} represents the smaller of x_p or x, x_l represents the larger of x_p or x, $\delta = 1$ for $x_p < x$, $\delta = -1$ for $x_p > x$, γ represents the complex propagation constant along the TL, ρ_1 and ρ_2 are the voltage reflection coefficients at the loads of the TL, h is TL height and L is length of TL.

The integrals of the Green's functions can be used to represent the solutions involving scattered voltage by (24) and (26).

$$V_s(x) = \int_0^L G_V(x;x_p)E_X\,dx_s + G_V(x;0)\int_0^h E_z^e(0,0,z)dz - G_V(x\,;\,L)\int_0^h E_z^e(L,0,z)dz \tag{24}$$

$$V_i(x) = -\int_0^h E_z^e(x,0,z)dz \tag{25}$$

LIV can be calculated by adding incident voltage (V_i) to scattered voltage (V_s) by (26).

$$\mathrm{LIV}(x) = V_s(x) + V_i(x) \tag{26}$$

2 Frequency-Dependent Models For The Soil Conductivity And Relative Permittivity

soil parameters as soil conductivity and soil permittivity are represented in case of non-frequency-dependent and frequency-dependent.

Used data in case of non-frequency dependent of soil parameters : σ_O = 0.001 S/m and $\mathcal{E}_{rg} = 10$.

The Alipio-Visacro Model and the Smith-Longmire Model are adopted for the investigations in this research to represent frequency dependent of soil parameters.

3.1 Smith-Longmire Model

Longmire and Longley proposed formulation for the soil. The expressions for relative permeability and ground conductivity are given by :

$$\mathcal{E}_{rg}(f)_{\textbf{Smith-Longmire Model}} = \mathcal{E}_\infty + \sum_{n=1}^{14} \frac{a_n}{1+\left(\frac{f}{\left(\left(\frac{p}{10}\right)^{1.28} * 10^{n-1}\right)}^{2}\right)^2} \tag{27}$$

$$\sigma_g(f)_{\textbf{Smith-Longmire Model}} = \sigma_0 + 2\pi\mathcal{E}_0 \sum_{n=1}^{14} \frac{a_n\left(\left(\frac{p}{10}\right)^{1.28} * 10^{n-1}\right)\left(\frac{f}{\left(\left(\frac{p}{10}\right)^{1.28} * 10^{n-1}\right)}\right)^2}{1+\left(\frac{f}{\left(\left(\frac{p}{10}\right)^{1.28} * 10^{n-1}\right)}\right)^2} \tag{28}$$

$$\sigma_o = 8 \times 10^{-3} \left(\frac{p}{10}\right)^{1.54} \left(\frac{\mathrm{S}}{\mathrm{m}}\right), \ \mathcal{E}_\infty = 5$$

Where a_n is a coefficient shown in Table I for various values of n, σ_o represents the low-frequency conductivity at 100 Hz, f represents the frequency ranging from dc to 5 MHz, $\mathcal{E}_{rg}(f)$ represents the relative permittivity, $\sigma_g(f)$ represents soil conductivity at each frequency, and p is known as the percentage of moisture soil and equal to 2.6%.

Table 1

a_n coefficient

n	1	2	3	4	5
a_n	3.4×10^6	2.74×10^5	2.58×10^4	3.38×10^3	5.26×10^2
n	6	7	8	9	10
a_n	1.33×10^2	2.72×10	1.25×10	4.8	2.17
n	11	12	13	14	
a_n	0.98	0.392	0.173	0	

3.2 Alipio-Visacro Model

Another soil model was proposed by Visacro and Alipio. They represent the soil characteristics' frequency dependency by the following :

$$\sigma_g(f)_{\textbf{Alipio-Visacro Model}} = \left[\sigma_o + \sigma_o h(\sigma_o)(\frac{f}{10^6})^{\gamma}\right] \times 10^{-3} \quad (29)$$

$$\mathcal{E}_{rg}(f)_{\textbf{Alipio-Visacro Model}} = \frac{\varepsilon'_{\infty}}{\varepsilon_o} + \frac{\tan\left(\frac{\pi\gamma}{2}\right).10^{-3}}{2\pi\varepsilon_o(10^6)^{\gamma}}\sigma_o h(\sigma_o) f^{\gamma-1} \quad (30)$$

where σ_{100Hz} is the value of σ_o [mS/m], $h(\sigma_o)$ =1.26 $\times\sigma_0^{-0.73}$and γ = 0.54.

4 Data of Manuscript Result

The effect of the ground losses and wire losses on Lightning induced voltage (LIV)considering both fixed model and two frequency-dependent models of soil parameters are studied. This study of ground losses effect is implemented with changing v (lightning velocity) as 40 m/μs, 120 m/μs, and 200 m/μs, changing h as 6 m and 10 m and changing d (distance between transmission line (TL) and lightning strike) as 50 m and 100 m. LIVs have been computed at two points on TL, which are the midpoint and 500 m from the midpoint of TL. The midpoint is the point on the line that is directly in front of the lightning channel. The results are compared with the FDTD method as shown in Appendix A in Manuscript.

Ultimately, we did not use any other data expect what we have mentioned in this document and mentioned also in the manuscript . Regarding the verification, we have proved our results with results are presented in this paper [5]. The main author of this paper is also the author of this manuscript if anyone needs to contact him. We did not use any data from this paper but just used it for verification of our manuscript result. All illustrations including figures and tables are mentioned in the text. To make sure, we highlight all figures and table in manuscript. Figure 13 is mentioned in the text from the first submission.
