# Supplementary material for: The impact of ground losses on estimations of lightning-induced voltage with dispersive soil parameters
Source: Sci Rep. 2025 Jul 6;15:24146. doi: 10.1038/s41598-025-08379-3 (PMC12230118; doi:10.1038/s41598-025-08379-3)
Supplement: Supplementary file 2 — Supplementary Information 2. [file 41598_2025_8379_MOESM2_ESM.pdf]

The impact of ground losses on estimations of lightning-induced voltage with dispersive soil parameters

Toqa Faisal[1,2] ✉. Sahar.S. Kaddah[1]. Taghreed Said[2]. Mohammad E.M. Rizk[1]

[1] Electrical Power and Machines Department, Faculty of Engineering, Mansoura University, Mansoura, Egypt. [2] Electrical Engineering Department, Higher Technological Institute,10th of Ramada City, Egypt. ✉ email: toqafaisal24@gmail.com.

Appendix A

Comparison of Our Methodology with the FDTD Method

Our methodology is compared with the FDTD method [5], as shown in Fig. A1. Figure A1 shows that the results obtained in the frequency domain align closely with those obtained using the FDTD method, both at the midpoint and at 500 m from the center of TL.

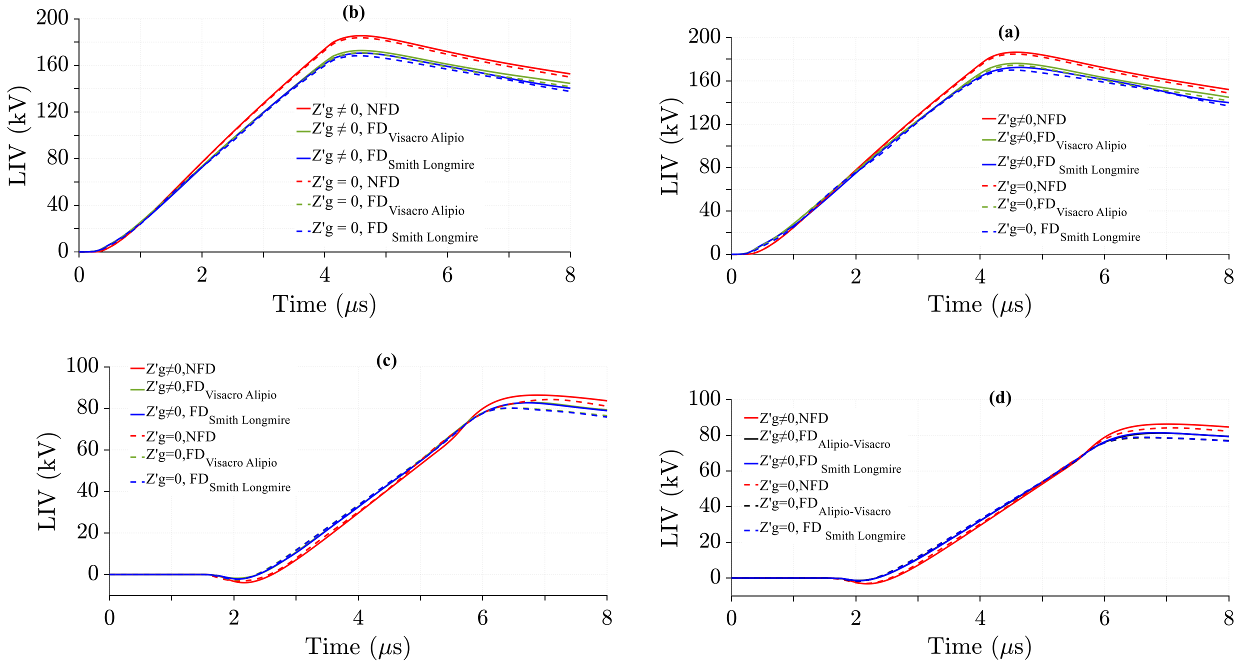

Fig. A1. LIV values using three soil models at d = 100 m, σ_o = 0.001 S/m, $\sigma_g(f)$ at p = 2.6%, ε_{rg} = 10, h = 10 m, and v = 120 m/ μs. (a), (b) at the midpoint; (c), (d) at 500 m from the midpoint. The first column [(a), (c)]: using CR formula in frequency domain. the second column [(b), (d)]: using the FDTD method.
